# Inter-Pregnancy Interval and the Incidence of Preterm Birth 

**Published:** 2020-03

**Authors:** Maryam Asgharnia, Tahereh Varasteh, Davoud Pourmarzi

**Affiliations:** 1 Reproductive Health Research Center, Guilan University of Medical Sciences, Rasht, Iran; 2 School of Public Health, Faculty of Medicine, The University of Queensland, Brisbane, Australia

**Keywords:** Inter-Pregnancy Interval, Preterm Birth, Preeclampsia, Gravidity

## Abstract

**Objective:** Preterm birth is associated with high rates of neonatal morbidity and mortality. This study aimed to investigate the relationship between inter-pregnancy interval and the incidence of preterm birth.

**Materials and methods:** In a case-control study, 185 women with preterm delivery and 185 women with term delivery were included. Data including inter-pregnancy interval, demographic characteristics, history of prenatal and neonatal complications, parity, gravidity, type of delivery, and smoking status were collected.

**Results:** The mean of the inter-pregnancy interval in the case and control groups were 79.84 ± 45.55 months and 78.49 ± 41.29 months, respectively (P = 0.767). Inter-pregnancy interval 12-month or less in comparison with Inter-pregnancy interval more than 24 months significantly increased the odds of preterm delivery (OR: 4.05, 95% CI: 1.06-15.39, p = 0.040). However, inter-pregnancy interval of 13-24 months was not a risk factor when compared with more than 24-month inter-pregnancy interval (OR: 1.54, 95% CI: 0.62-3.80, p = 0.351). Having an educational level less than high school in comparison with tertiary level decreased the odds of preterm delivery (OR: 0.25, 95% CI: 0.11-0.56, P = 0.040). With each increase in number of gravidity odds of preterm delivery increased by 1.5 times (95% CI: 1.11-2.04, P = 0.009). Having a history of preterm delivery (OR: 2.57, 95% CI: 1.17-5.64, P = 0.019) and experiencing preeclampsia (OR: 1.98, 95% CI: 1.06-3.68, P = 0.032) increased the odds of preterm delivery.

**Conclusion:** Inter-pregnancy interval of 12-month or less in comparison with more than 2-year inter-pregnancy interval, experiencing preeclampsia, history of preterm delivery and increased number of gravidity increase the risk of preterm delivery. Health care providers need to be informed with the appropriate inter-pregnancy interval and counsel women to make an informed decision regarding their pregnancy.

## Introduction

According to World Health Organization (WHO) definition, preterm birth is defined as births less than 37 completed weeks of gestation (1). Preterm birth is associated with high rates of neonatal complications. Preterm birth complications are the leading cause of neonatal death and the second most frequent cause of death in children under 5 years after pneumonia (2-4). Worldwide, approximately 11% of births are affected by preterm delivery (3). The prevalence of preterm delivery in Iran was estimated at 9.2 (95% CI: 7.6-10.7) (5). 

Although there have been several risk factors for preterm delivery, about two-third of preterm delivery cannot be attributed to any risk factors (3). The reported risk factors include a history of preterm delivery, premature rupture of the membrane, anemia, low socioeconomic status, smoking, multiple gestation and short inter-pregnancy interval (3, 6). While many studies have found that there is a relationship between inter-pregnancy interval and the adverse outcomes of preterm delivery, there is no consistency among studies in definition of appropriate inter-pregnancy interval (6-13). 

The effect of both short and long inter-pregnancy intervals on maternal and neonatal complications are considered. To explaining the effects of short inter-pregnancy interval, maternal reduction hypothesis suggests insufficient maternal recovery from the physiological stress of pregnancy and lactation (14). Maternal folate deficiency, hormonal imbalance and postpartum nutritional stress have also been suggested to explain these effects (14). A mechanism has also been proposed to justify the effects of long inter-pregnancy interval indicating that the benefits of previous births, such as maternal physiological adaptation gradually diminish and returns the mother to conditions of a primipara woman, which is known as the physiological regression hypothesis (15). These theories emphasize the need for an appropriate inter-pregnancy interval which gives mothers enough time to recover from last pregnancy, but not that long that reduces the mothers’ compatibility (15). Considering these theories, it seems that the appropriate inter-pregnancy interval can be different in different socioeconomic and ethnic groups (14, 15).

In Iran, small number of studies have investigated inter-pregnancy interval. Moreover, studies that have considered the inter-pregnancy interval as a risk factor for preterm delivery were cross-sectional in design in most cases (5). Therefore, case control study may help better understand the effect of inter-pregnancy intervals on the incidence of preterm delivery. This case-control study aimed to examine the relation between inter-pregnancy interval and the incidence of preterm delivery after the elimination of confounding variables.

## Materials and methods

In a case-control study, 185 women with preterm delivery at 22 to 37 weeks of gestation (case group) and 185 women with term delivery after 37 weeks of gestation (control group) were included. Participants were recruited from a prenatal clinic at Al-Zahra Hospital, Rasht, Iran between May 2016 and February 2017. Women with labor induction, twin or multiple births, post term delivery, primipara women (Gravid I), and women who had a previous abortion before 22 weeks pregnancy were excluded.

Data including age, pre-pregnancy BMI, weight, height, educational level, place of residence, inter-pregnancy intervals, gestational diabetes, preeclampsia, history of stillbirth, history of preterm delivery, history of giving birth to a low weight infant, parity (number of the birth), gravidity (the number of pregnancy), smoking and placenta position were collected through interview with participants and from mothers recorded data at the hospital prenatal clinic

***Statistical analysis***: Independent t test was used to compare means between case and control groups. Chi-square or Fisher's exact tests were used to compare the distribution of study participants in each category of categorical variables between case and control groups.

***Ethical consideration*** ***: ***This study was approved by the Ethics Committee of Guilan University of Medical Sciences (IR.GUMS.REC.1394.466). Written informed consents were obtained from all participants before enrollment in the study.

## Results

The mean of inter-pregnancy interval in the case group was 79.84 ± 45.55 months and in the control group was 78.49 ± 41.29 months (p = 0.767). There was no significant difference between the two groups in terms of age, maternal pre-pregnancy weight, pre-pregnancy BMI, and parity. The mean number of gravidity in the case group (2.56 ± 0.84) was higher than control group (2.35 ± 0.71) (p = 0.010). Only one woman with term delivery was smoker (Table 1).

In case group, the percentages of women in inter-pregnancy interval categories of “less than or equal to 12 months” and “13-24 months” were higher compare to control group, and this percentages in category “more than 24 months” was lower than compare to control group (p = 0.048). There were significant differences between case and control groups in terms of level of education (p < 0.001), place of residence (p = 0.009), experiencing preeclampsia, (p = 0.005) and having a history of preterm delivery, (p = 0.001). There were no significant differences between the two groups in terms of experiencing gestational diabetes (p = 0.678), history of stillbirth (p = 0.685), history of labor with low birth weight (p = 0.836), and placenta position (p = 0.125) (Table 1).

**Table 1:** Comparison of studied variables between case and control groups

**Characteristics**		**Case group (n = 185)**	**Control group (n = 185)**	**P-value**
Inter-pregnancy interval (month)		79.84 ± 45.75	78.49 ± 41.29	0.767
Age (year)		31.75 ± 5.01	31.41 ± 4.70	0.501
Pre-pregnancy weight (kg)		70.20 ± 14.78	70.25 ± 14.01	0.971
Pre-pregnancy BMI (kg/m^2^)		27.30 ± 5.31	27.06 ± 5.16	0.658
Parity		2.24 ± 0.84	2.25 ± 0.53	0.881
Gravidity		2.56 ± 0.84	2.35 ± 0.71	0.010
Inter-pregnancy interval	≤ 12 months	11 (5.9)	3 (1.6)	0.048
	13-24 months	15 (8.1)	10 (5.4)	
	More than 24 months	159 (85.9)	172 (93)	
Level of education	Illiterate	2 (1.08)	2 (1.08)	0.001*
	Primary and middle school	58 (31.3)	96 (51.8)	
	High school and diploma	98 (52.9)	76 (41.08	
	Tertiary education	27 (14.59)	11 (5.94)	
Place of residence	Urban area	131 (70.81)	107 (57.84)	0.009*
	Rural area	54 (29.19)	78 (42.16)	
Gestational diabetes	Yes	30 (16.2)	33 (17.8)	0.678*
	No	155 (83.7)	152 (82.1)	
Preeclampsia	Yes	40 (21.6)	20 (10.8)	0.005*
	No	145 (78.3)	165 (89.1)	
History of stillbirth	Yes	4 (2.1)	2 (1.08)	0.685**
	No	181 (97.8)	183 (98.9)	
Preterm labor history	Yes	31 (16.7)	10 (5.4)	0.001*
	No	154 (83.2)	175 (94.5)	
History of low birth weight delivery	Yes	13 (7.02)	12 (6.4)	0.836*
	No	172 (92.9)	173 (93.5)	
Placenta position	Normal	174 (94.05)	180 (97.2)	0.125*
	Abnormal	11 (5.9)	5 (2.7)	

Data are presented as Mean ± SD or number (%).

* chi square,

** Fisher's Exact Test

Results from logistic regression showed inter-pregnancy interval 12 month or less in comparison with inter-pregnancy interval more than 24 months significantly increased the odds of preterm delivery (OR: 4.05, 95% CI: 1.06-15.39, p = 0.040). However, inter-pregnancy interval of 13-24 months was not a risk factor when compared with more than 24-month inter-pregnancy interval (OR: 1.54, 95% CI: 0.62-3.80, p = 0.351). 

Having an educational level less than high school in comparison with tertiary education decreased the odds of preterm delivery (OR: 0.25, 95% CI: 0.11-0.56, p = 0.040). With each increase in number of gravidity odds of preterm delivery increased by 1.5 times (95% CI: 1.11-2.04, p = 0.009). Having a history of preterm delivery (OR: 2.57, 95% CI: 1.17-5.64, p = 0.019) and experiencing preeclampsia (OR: 1.98, 95% CI: 1.06-3.68, p = 0.032) increased the odds of preterm delivery (Table 2).

**Table 2:** Results of logistic regression analysis on risk factors of preterm delivery

**Variable**		**OR (95% CI)**	**P-value**
Inter- pregnancy interval	More than 24 months (n = 331)	1	-
	13 to 24 months (n = 25)	1.54 (0.62-3.80)	0.351
	12 months and less (n = 14)	4.05 (1.06-15.39)	0.040
Level of Education	Tertiary Education (n = 38)	1	-
	High school and Diploma (n = 174)	0.53 (0.24-1.17)	0.351
	Less than high school (n = 158)	0.25 (0.11-0.56)	0.040
Gravidity		1.50 (1.11-2.04)	0.009
History of preterm delivery		2.57 (1.17-5.64)	0.019
Preeclampsia		1.98 (1.06-3.68)	0.032

## Discussion

Based on the results of this study, inter-pregnancy interval 12-month or less in comparison with inter-pregnancy interval more than 24-month increased the odds of preterm delivery by 4 times. Women with inter-pregnancy interval of 13-24 months had not higher risk of preterm delivery when compared to women with inter-pregnancy interval more than 24-month. Although this finding is affected by small number of women in the category of 12-month or less inter-pregnancy interval, previous studies showed the risk of preterm delivery in women with short inter-pregnancy interval (6-13). In Ball’s study it has been reported that the inter-pregnancy interval less than 18 months and also longer than 23 months can increase the risk of preterm birth (8). In another study the interval less than 6 months in comparison with 18-23 months increased the risk of preterm delivery (10). Sufficient inter-pregnancy interval is needed for mothers to recover from the previous pregnancy (6). Maternal reduction hypothesis suggests insufficient maternal recovery from the physiological stress of pregnancy and lactation can lead to adverse outcomes including preterm delivery (14). Considering the adverse outcomes of preterm birth and inter-pregnancy interval as its modifiable risk factor (4), educational programs for population and health care providers need to emphasis the importance of at least 12 months interval between pregnancies.

In this this study, the history of preterm delivery tripled the odds of preterm delivery. In other study it has been shown that women who had previous preterm birth are in higher risk of preterm delivery (16, 17). Health care providers need to consider this factor in assessment and management of pregnant women and provide needed care to prevent preterm delivery. 

This study found that the experiencing preeclampsia increased the odds of preterm delivery. This is also evident from previous study in Iran (18). In our study, severe and mild preeclampsia was not categorized separately. Preeclampsia itself, if severe, can be a reason for preterm delivery. High blood pressure can increase the resistance of uterus vessels and reduce the uterine-peritoneal fluid, which causes Intrauterine growth restriction (19).

Based on our findings, in comparison with women with academic education, women with educational level lower than high school had lower odds of preterm delivery. However, women with educational level high school and diploma were not at higher risk of preterm delivery when compared with women with academic education. This finding is inconsistent with findings from other studies (20). The level of education can be closely related to the mother's occupation and socioeconomic status which play important roles in preterm delivery. In this study we did not collected data on mothers’ occupation and are not able to clarify this relationship. 

Our findings showed that with increase in number of gravidity, the odds of preterm delivery increased significantly. In Alijahan’s study in Iran, no difference between multigravida and primigravid women in terms of the preterm delivery incidence was reported (18). Studies’ findings related to this factor are inconsistent. In some studies, the increased number of gravidity was associated with increased risk of preterm delivery whereas in other studies no effect or protective effect of increased number of gravidity was reported. It needs to be considered that in this study we excluded primipara women which were included in other studies. (21-23).

***Strength and Limitations: ***In this study we used a case-control design which enabled us to examine various risk factors for preterm delivery. Some data were collected through interview with participants which recall bias may be an issue. The study sample were women who attended a teaching hospital in Rasht which may not be a representative sample of all pregnant women in Iran.

## Conclusion

Based on the findings of this study, inter-pregnancy interval of 12-month or less in comparison with more than 2-year inter-pregnancy interval increases the risk of preterm delivery by 4-fold. Also, women with a history of preterm delivery and those who had experienced preeclampsia in their current delivery had a greater risk preterm delivery. With consideration of inter-pregnancy interval as a modifiable risk factor for preterm delivery, educational programs during prenatal visits and after the delivery are recommended. Health care providers especially midwives, obstetricians and general practitioners need to be informed with the appropriate inter-pregnancy interval and counsel women to make an informed decision regarding their pregnancy.

## References

[1] World Health Organization Preterm birth, Fact sheet: World Health Organization: 2018.

[2] Liu L, Oza S, Hogan D, Perin J, Rudan I, Lawn JE (2015). Global, regional, and national causes of child mortality in 2000–13, with projections to inform post-2015 priorities: an updated systematic analysis. Lancet.

[3] Vogel JP, Chawanpaiboon S, Moller A-B, Watananirun K, Bonet M, Lumbiganon P (2018). The global epidemiology of preterm birth. Best Pract Res Clin Obstet Gynaecol.

[4] Beck S, Wojdyla D, Say L, Betran AP, Merialdi M, Requejo JH (2010). The worldwide incidence of preterm birth: a systematic review of maternal mortality and morbidity. Bull World Health Organ.

[5] Vakilian K, Ranjbaran M, Khorsandi M, Sharafkhani N, Khodadost M (2015). Prevalence of preterm labor in Iran: a systematic review and meta-analysis. Int J Reprod Biomed (Yazd).

[6] DeFranco EA, Stamilio DM, Boslaugh SE, Gross GA, Muglia LJ (2007). A short interpregnancy interval is a risk factor for preterm birth and its recurrence. Am J Obstet Gynecol.

[7] Wendt A, Gibbs CM, Peters S, Hogue CJ (2012). Impact of increasing inter‐pregnancy interval on maternal and infant health. Paediatr Perinat Epidemiol.

[8] Ball SJ, Pereira G, Jacoby P, De Klerk N, Stanley FJ (2014). Re-evaluation of link between interpregnancy interval and adverse birth outcomes: retrospective cohort study matching two intervals per mother. BMJ.

[9] Smits LJ, Elzenga HM, Gemke RJ, Hornstra G, van Eijsden M (2013). The association between interpregnancy interval and birth weight: what is the role of maternal polyunsaturated fatty acid status?. BMC pregnancy childbirth.

[10] Zhu BP (2005). Effect of interpregnancy interval on birth outcomes: findings from three recent US studies. Int J Gynaecol Obstet.

[11] Howard EJ, Harville E, Kissinger P, Xiong X (2013). The association between short interpregnancy interval and preterm birth in Louisiana: a comparison of methods. Matern Child Health J.

[12] Grisaru-Granovsky S, Gordon ES, Haklai Z, Samueloff A, Schimmel MM (2009). Effect of interpregnancy interval on adverse perinatal outcomes-a national study. Contraception.

[13] Simonsen S, Lyon JL, Stanford JB, Porucznik CA, Esplin MS, Varner MW (2013). Risk factors for recurrent preterm birth in multiparous Utah women: a historical cohort study. BJOG.

[14] Miller JE (1991). Birth intervals and perinatal health: an investigation of three hypotheses. Fam Plann Perspect.

[15] Zhu BP, Rolfs RT, Nangle BE, Horan JM (1999). Effect of the interval between pregnancies on perinatal outcomes. N Engl J Med.

[16] van den Broek NR, Jean-Baptiste R, Neilson JP (2014). Factors associated with preterm, early preterm and late preterm birth in Malawi. PloS one.

[17] do Carmo Leal M, Esteves-Pereira AP, Nakamura-Pereira M, Torres JA, Theme-Filha M, Domingues RMSM (2016). Prevalence and risk factors related to preterm birth in Brazil. Reproductive health.

[18] Alijahan R, Hazrati S, Mirzarahimi M, Pourfarzi F, Hadi PA (2014). Prevalence and risk factors associated with preterm birth in Ardabil, Iran. Iran J Reprod Med.

[19] Sharma D, Shastri S, Sharma P (2016). Intrauterine growth restriction: antenatal and postnatal aspects. Clin Med Insights Pediatr.

[20] Ruiz M, Goldblatt P, Morrison J, Kukla L, Švancara J, Riitta-Järvelin M (2015). Mother's education and the risk of preterm and small for gestational age birth: a DRIVERS meta-analysis of 12. J Epidemiol Community Health...

[21] Ghose ST, Samal S, Armugam S, Parida P (2014). Measurement of cervical biometry using transvaginal ultrasonography in predicting preterm labor. J Nat Sci Biol Med.

[22] Ahankari A, Bapat S, Myles P, Fogarty A, Tata L (2017). Factors associated with preterm delivery and low birth weight: a study from rural Maharashtra, India. F1000Res.

[23] Wang H, Hu YF, Hao JH, Chen YH, Wang Y, Zhu P (2016). Maternal serum zinc concentration during pregnancy is inversely associated with risk of preterm birth in a Chinese population. J Nutr.

